# Technical Efficiency and Organ Transplant Performance: A Mixed-Method Approach

**DOI:** 10.3390/ijerph120504869

**Published:** 2015-05-05

**Authors:** Carmen de-Pablos-Heredero, Carlos Fernández-Renedo, Jose-Amelio Medina-Merodio

**Affiliations:** 1 School of Social Sciences, Universidad Rey Juan Carlos, PO Box 28032, Madrid 28032, Spain; 2 Castilla and Leon Transplant Coordination Office, National Health System, León 24071, Spain; E-Mail: cfernandezr@saludcastillayleon.es; 3 Computer Science Department, University of Alcalá, Alcalá de Henares, Madrid 28871, Spain; E-Mail: josea.medina@uah.es

**Keywords:** mixed methods research, organ transplant, technical efficiency, Baldrige indicators, donation and transplant processes

## Abstract

Mixed methods research is interesting to understand complex processes. Organ transplants are complex processes in need of improved final performance in times of budgetary restrictions. As the main objective a mixed method approach is used in this article to quantify the technical efficiency and the excellence achieved in organ transplant systems and to prove the influence of organizational structures and internal processes in the observed technical efficiency. The results show that it is possible to implement mechanisms for the measurement of the different components by making use of quantitative and qualitative methodologies. The analysis show a positive relationship between the levels related to the Baldrige indicators and the observed technical efficiency in the donation and transplant units of the 11 analyzed hospitals. Therefore it is possible to conclude that high levels in the Baldrige indexes are a necessary condition to reach an increased level of the service offered.

## 1. Introduction

Organ transplant systems are in need of fundamental changes if they are to provide increasing levels of service delivery under more demanding circumstances and severe budget constraints. Current organ transplant structures do not make the best use of their resources resulting in suboptimal care processes and resource overuse [1,2].

In response to the challenge of how to best optimize the resource utilization in organ transplant processes, there is an increasing interest, according to published literature, in the analysis of productivity and efficiency achieved in health care systems [3,4].

With regards to this challenge two are the main purposes of this paper: (1) to provide formal methods to conduct quantitative appraisal of the performance observed in health care delivery systems and (2) to further investigate the effect of systems processes and internal structures on this performance.

The organ donation process constitutes an interesting case of study in a triangulation approach as it entails a very delicate and complex set of processes involving several highly specialized experts working in disparate organizations with rather different structures and resources. It demands lots of resources that must be efficiently managed.

With regards to the existing limitations observed in parametric models, this paper develops an approach to compute technical efficiency providing enough flexibility in the model structure to allow for: (1) multi-output analysis of technical efficiency as well as (2) the modeling of complex data structures present in longitudinal observations or hierarchical business units in health care.

With regards to the second purpose of the paper, understand the impact of internal processes within health care units in the observed performance, a qualitative research study was conducted whereby each service unit was characterized based on the Baldrige criteria.

The rest of the paper is structured as follows: Section 2 provides a formal method to conduct quantitative analysis of technical efficiency in a set of organ donation and transplant service units. Section 3 characterizes organizational routines and processes within those service units. Section 4 builds upon previous sections to build causal models explaining the relationship between organizational routines and observed performance and Section 5 offers the paper’s conclusions.

The present analysis can help to promote organizational characteristics that offer the best decision making in the organ transplant process and therefore to optimize the resource utilization in organ transplant processes.

## 2. Theory Development

Organ transplant systems are adaptive because, unlike mechanical systems, they are composed of individuals—patients and clinicians—who have the capacity to learn and change as a result of experience. Their actions in delivering organs are not always predictable, and tend to change both their local and larger environments. The unpredictability of behavior in complex adaptive systems can be seen as contributing to huge variation in the delivery of health care [5].

This calls for systemic approaches in which health care services follow a Service-Dominant (S-D) logic [6,7]. Organ donation, an instance of this type of healthcare servicing, fits in the foundational premises for S-D logic as suggested in Vargo and Akaka [8] in the sense that organ donation is a service in which some competences, donation capabilities, are applied for the benefit of others. There exists an indirect service exchange between provider and adopter mediated by hospitals and physicians. Operant resources, such as surgeons, are key to the service in contrast to operand resources which play a secondary role in determining effectiveness [9].

Value is always co-created in organ donation processes by several parties engaged in close collaboration, each of them offering a specific core competence [10]. There is no single entity fully responsible for value delivery. Customers, in this case experts at the receiving hospital, play an active role by deciding under which conditions a donation is to be performed according to medical characteristics. In most cases, they even travel to the donating hospital to harvest organs by themselves.

In the following discussions the organ donation service will be described in terms of two basic constructs in S-D logic: service systems and service interactions occurring between them. It is increasingly evident that patient outcomes are not solely a function of efficacious clinical interventions and practices. Delivery-system research may be viewed as the systematic study of healthcare organizations, including interchanges with their external environments (e.g., markets, regulators, competitors) and interactions among internal components (e.g., employees, technology, work processes, culture), that affect how care is organized and provided [1].

The difficulty in the design of the organ transplant process where many different specialists take part has much to do with the way the transfer of knowledge is provided [11,12] and in structures where the tacit component is high [13,14,15] in contexts where there is a clear description of the structures and results [16]. Besides, for the structure designs to be efficient, they must be able to adapt to new scenarios and dynamics [17]. This way they will be aligned with the evolution of scientific and technological possibilities by maintaining optimal response time standards.

Besides, the need to maintain and generate dynamic capabilities in system processes together with the need to manage tacit knowledge, difficult to turn explicit, makes necessary the use of coordination mechanisms that exceed excellent organizational routines and that warranty the proper climate to promote interdependent and multidisciplinary processes [18].

Drucker [19] considers that one of the main elements to incentivize productivity and performance in structures is the association of people that work together for similar purposes. Pisano [20] suggests that although there are no universal formulas to favor the transfer of knowledge and learning, the existent ones depend each time more on the organizational structures. A group of authors [21,22,23] suggests that the link between incremental uncertainty and more informal coordination models drives to the achievement of higher performance in system processes.

Technical efficiency represents the ability of the observed health care unit to maximize the results of the service delivery subject to some resources and constraints, or conversely the ability to maintain the service delivery with lower levels of resource consumption.

Seminal papers on the measurement of technical efficiency like [24] and [25] stated the importance of productive efficiency for policy and economic planning purposes. Twenty years later [26] introduced Data Envelopment Analysis (DEA), a methodology able to assess the relative efficiency of multi-input multi-output production units.

At its most basic form DEA computes technical efficiency scores as descriptive measures of the relative technical efficiency of observed decision making units in comparison to a best-practice production frontier. Being of a non-parametric nature DEA does not impose any functional form on the production model, arguably a key aspect for its widespread application in a variety of contexts from banking [27,28,29], production planning [30], R&D performance [31,32] and agricultural economics [33] among others [34].

In some circumstances it might be of interest to ask whether observed firms can improve their importance and if so, by how much. According to [35], such questions can only be answered by inference which in order to be meaningful requires coherent, well-defined statistical models.

The majority of the results presented in the reviewed literature adopting parametric models to conduct efficiency analysis adopt however model structures which are too simplistic, e.g., conventional ordinary least square regression models, to characterize the rich variety present in health care deliveries [36,37,38,39,40,41,42]. Moreover, conventional parametric models do not allow for multi-output response analysis, arguably an important limitation in the analysis of complex, multidimensional services.

With regards to the existing limitations observed in parametric models, this paper follows a parametric multilevel modeling approach to compute the technical efficiency achieved by the service delivery systems under study.

For example, hierarchical linear models (HLMs) offer a powerful approach to conduct longitudinal analyses across three or more levels [39]. These models are also commonly known by other names, such as mixed-effects regression models and multilevel models [37,42]. They are able to recognize the hierarchical structures present in complex service delivery systems.

The Baldrige quality criteria have been applied to stimulate actions for total quality management at firms and analyze the results [43,44,45]. In 1998 the criteria were extended in the United States to health services [46].

**Figure 1:**
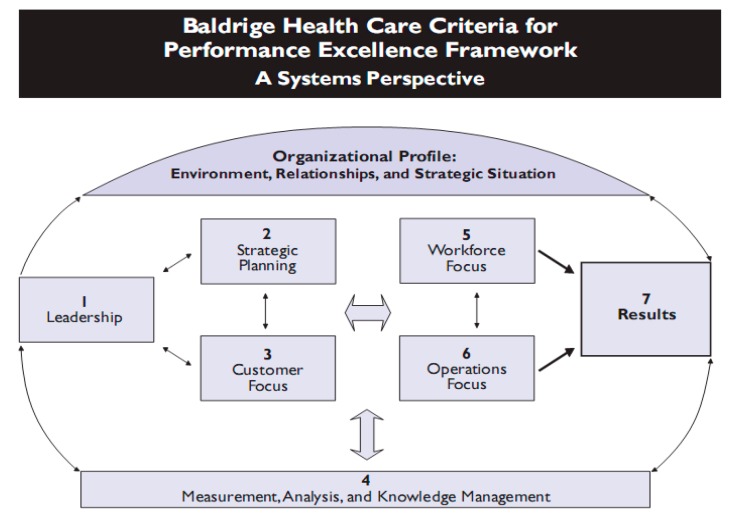
The Baldrige model.

The framework for Baldrige criteria for excellence in performance presents a managerial model based on the quality [43] and the orientation to an effective performance to improve processes [47]. The model is organized in seven interrelated components: (1) leadership, (2) focus on customers and the rest of interest groups, (3) strategic planning (4) the management of human resources (5) the management of the information and data analysis, (6) the management of processes (7) and the management of results for final performance. Many health institutions today are using Baldridge’s criteria as a tool to self-evaluate the effects of the quality practices in their organizations [48]. Figure 1 offers a conceptualization of the Baldrige criteria. To achieve quality in the delivery of healthcare services the following are needed:

1. First being able to design and implement processes in the structures.
2. To reach levels of better coordination associated to the system processes.

According to the previous literature review, the following hypotheses are proposed:

H1 To reach best results, processes must be oriented to the achievement of objectives

H2 More efficient organizations in terms of coordination reach best results

H3 High levels of organizational routines and processes positively moderate the resulting technical efficiency.

## 3. Research Methodology

The theoretical approach followed in the previous section adopts a systems perspective, however this raises a number of methodological challenges in the context of delivery-systems interventions as purely quantitative approaches are not sufficient to capture the richness of the context in which the service delivery takes place [49].

The effect that internal system processes and structures exert in observed performance is mediated by a range of human, socio-cultural and organizational factors collectively referred as the context. Therefore any research in progress needs to take into account the situational opportunities and constraints that affect the occurrence and meaning of organizational activities [50,51].

As far as research design is concerned this paper adopts a mixed method research process in the sense of combining in the research process elements of qualitative and quantitative research in order to achieve breadth and depth of understanding and corroboration [51].

Evidence in the published literature attests to the current use of mixed methods approaches in health-related research, such as in cardiology [52], pharmacy [53], family medicine [54], pediatric oncology nursing [55], mental health services [56,57], disabilities [58], and public health nutrition [59].

### 3.1. Contextualization of Organ Donation and Transplant Delivery Services

Contextualization is the process whereby knowledge of the settings to be studied is brought to bear in conceptualization, research design, and implementation decisions. In this sense organ donation and transplant services present several important differences compared to other more conventional healthcare service delivery systems. In the first place the strict timing constraints call for high levels of coordination among the involved stakeholders [18].

Contrary to other knowledge-intensive activities, organ donation systems involve a strong component of variability of the contexts under which some of the donor-service systems need to perform. Organ transplant is a co-creative undertaking that is specific to each situation depending not only on the donor, but at the same time on the recipients’ characteristics [60]. This precludes complete standardization and decision making requires high levels of expertise and the autonomy of experts, in this case surgeons [14].

### 3.2. Mixed Method Design

**Figure 2:**
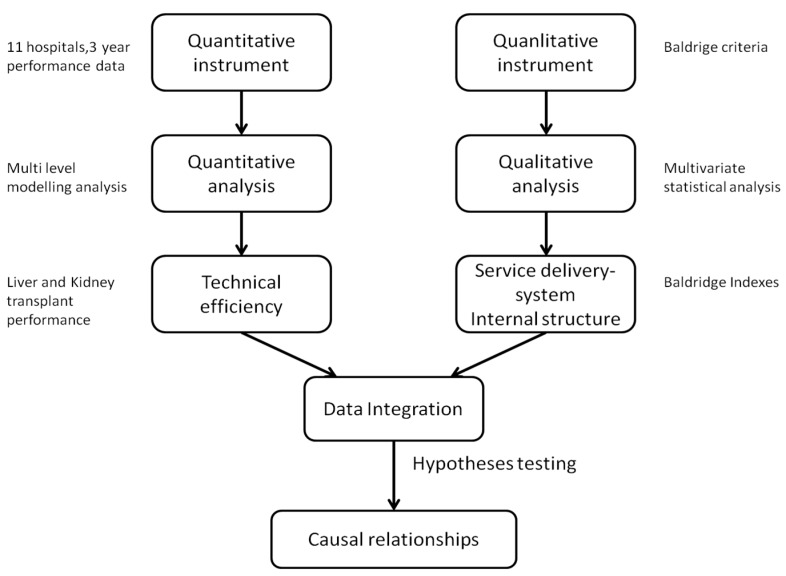
The research perspective.

The nature of the hypotheses formulated in Section 2 require a dual research approach combining qualitative and quantitative methods of inquiry in order to develop a complete understanding of the influence of system processes and structures in the observed performance in organ donation and transplant services. The research follows a sequential mixed method design whereby an initial quantitative exploration is followed by several qualitative analyses aimed at explaining in more depth the mechanisms underlying the phenomena under observation [61]. Figure 2 provides an overall perspective of the research design adopted in this paper.

## 4. Results

### 4.1. Quantitative Analysis of Technical Efficiency in Organ Transplant

#### 4.1.1. Multi-Output, Multilevel Technology Functions

Many kinds of data, including observational data collected in biological and managerial sciences have a hierarchical or clustered structure. For example service delivery units within a given hospital will exhibit more similar characteristics than units from other hospitals.

We refer to a hierarchy as consisting of units grouped at different levels. Hence service delivery units may be the level 1 units in an n-level structure where the level 2 units are hospitals in turn aggregated in a level 3 regional health care system.

The existence of such data hierarchies is neither accidental nor ignorable as doing so risks overlooking the importance of the group effects in the analysis and oftentimes renders invalid many of the traditional parametric techniques to the analysis of technical efficiency [28]. An important example of hierarchically structured data occurs when the same individuals or units are measured on more than one occasion. In this case occasions are clustered within individuals that represent level 2 units with level 1 unit measurement occasions.

This paper adopts multilevel generalized linear models as the framework to define technology functions as they: (1) provide sufficient flexibility to model clustered structures; (2) allow for non-linear models, e.g., arising in the case of censored variables or count models; (3) allow for the computation of technical efficiency in the case of several response variables. Conventional parametric techniques are not able to compute the technical efficiency of a combined set of outputs; in this sense this paper provides an interesting extension to the applicability of parametric techniques.

Appendix Table A1 presents a panel data corresponding to 11 hospitals involved in the Spanish system for organ donation and transplant for the period 2008–2010 [62]. Every hospital consisted of a set of service units in charge of: (1) finding adequate donors for potential transplants (2) perform the required medical processes and (3) conduct organ transplants [63]. The panel data represents two outputs: kidney and liver transplants.

These transplants are conducted by hospital “id” during the period “year”. Three inputs are considered, the total number of donors, the type of hospital “unittype” and the amount of donors above 70 years of age “donors70_100”.

The following table presents the mean and the standard deviation of the two outputs considered: kidney transplant and liver transplant. According to the data a clear dependence exists among the type of hospital and the moments of the output, this is so as hospitals with advanced technologies (codified with the *unittype* variable set to 2) are able to conduct more transplants, and also because advanced hospitals correspond to large cities with larger populations. The data from the Table 1 lead us to consider output variables following Poisson distributions albeit with some levels of overdispersion.

**Table 1:** The output variables.

Unittype		Summary: Kidney Response			Summary: Liver Response			
			Mean	Standard Dev.	N	Mean	Standard Dev.	N
**0**	**Basic transplant services**		5.62	4.1	24	3.1	2.5	24
**1**	**Neurological services**		29	7.2	8	16.4	4.6	8
**2**	**Neurological and advanced transplant services**		23.4	11.1	12	13.3	6.8	12

The large variety in the observed responses, kidney and liver transplants, between hospitals and the levels of over dispersion, *i.e.*, the variance is larger than the mean, rendering conventional parametric models useless. Moreover we are interested in finding the technical efficiency of each hospital for the combined response of the two outputs: kidney transplant and liver transplant and this calls for multilevel technology functions.

**Figure 3:**
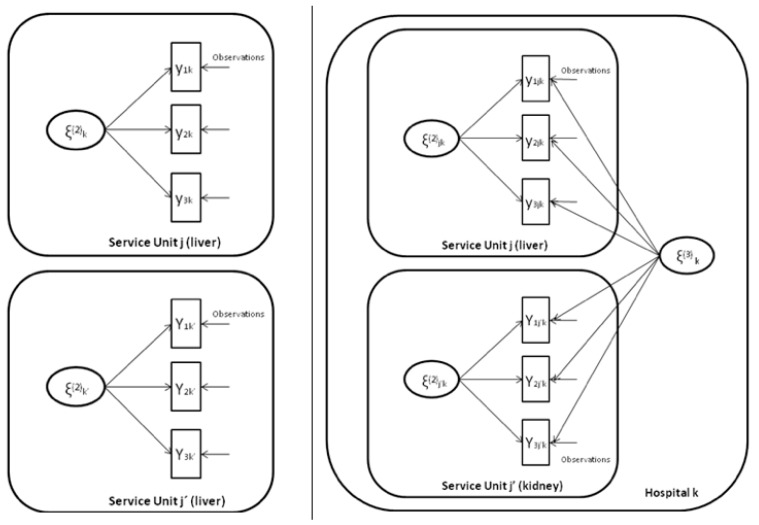
Technology functions (single-output and multi-output).

The following Figure 3 represents two different alternatives for technology functions. On the left a two-level model is assumed whereby observations (1st level i) are clustered into service units (2nd level j) where${\text{ ζ}}_{\text{k}}^{\left(2\right)}$ is the random effect capturing the ability of each service unit to perform (e.g., kidney transplants). On the right hand side a three-level model is built upon the previous one, this time each service unit is clustered into hospitals (3rd level k). This three-level model therefore implements a multi-output technology function that allows for the computation of the technical efficiency corresponding to the ability of each hospital to perform as a whole taking into account all service units combined (e.g., kidney and liver transplants). Formally speaking this “ability” corresponds to the technical efficiency as represented by the random effect$\;{\text{ζ}}_{1\text{k}}^{\left(3\right)}$.

Therefore we entertain a technology function of the form:

(1) $$\nu =\beta_{1}+\beta_{2}x_{2i}+\beta_{3}x_{3ik}+\cdots +{\text{ζ}}_{1\text{k}}^{\left(3\right)}+{\text{ζ}}_{2\text{k}}^{\left(2\right)}x_{2ik}$$

(2) $$liver\;response:\;y_{ij}\to Poisson\left(\mu_{ij}\right)$$

(3) $$kidney\;response:\;y\prime_{ij}\to Poisson\left(\mu \prime_{ij}\right)$$

Imposing a log-link relationship the technology function becomes:

(4) $$\begin{array}{c} \text{Ln}\left(\mu_{ij}+\delta \cdot {\mu^{\prime }}_{ij}\right)=\beta_{1}+\beta_{2}x_{2i}+\beta_{3}x_{3ik}+\cdots +{\text{ζ}}_{1\text{k}}^{\left(3\right)}+{\text{ζ}}_{2\text{k}}^{\left(2\right)}x_{2ik}=\\ =(\beta_{1}+{\text{ζ}}_{1\text{k}}^{\left(3\right)})+(\beta_{2}+{\text{ζ}}_{2\text{k}}^{\left(2\right)})x_{2i}+\beta_{3}x_{3ik}+\cdots + \end{array}$$

The previous expression (4) corresponds to a multi-output random-coefficient Poisson regression model accommodating: (1) two different responses: liver and kidney transplants; (2) dependence among the repeated observations; and (3) dependence among different service units within the same hospital, refer to Figure 3.

It is assumed that random effects are of the form ${\text{ζ}}_{1\text{k}}^{\left(3\right)}∽\text{N}\left(0,{\text{ψ}}_{11}^{\left(2\right)}\right)$ and ${\text{ζ}}_{2\text{k}}^{\left(2\right)}∽\text{N}\left(0,{\text{ψ}}_{22}^{\left(2\right)}\right)$ with covariance ${\text{ψ}}_{21}^{\left(2\right)}$. Expression (reference to likelihood) is computed via [64].

For the data presented in Appendix Table A1, the Table 2 presents the computed estimates for different technology functions: (1) multi-output three-level kidney and liver; (2) single-output two-level kidney; and (3) single-output two-level liver.

**Table 2:** Computed estimates for different technology functions.

		Conditional Effects: Combined Responses		Conditional Effects: Independent Responses			
		RC-Poisson: Multi-Output		RC-Poisson: Kidney		RC-Poisson: Liver	
		Est	(95% CI)	Est	(95% CI)	Est	(95% CI)
**Fixed Part: rate ratios**							
**exp(β_2_)**	**(unittype)**	1.21 ***	(1.08,1.36)	1.15 ***	(1.04,1.27)	1.15 **	(1.00,1.31)
**exp(β_3_)**	**(donors)**	1.10 ***	(1.08,1.12)	1.10 ***	(1.07,1.12)	1.09 ***	(1.07,1.11)
**exp(β_4_)**	**(donors 70–100)**	0.96 **	(0.94,0.99)	0.95 ***	(0.92,0.98)		
**Random Part**							
$\psi_{11}^{\left(3\right)}$			0.17		0.19		0.01
$\psi_{22}^{\left(2\right)}$			0.026		0.0017		0.002
$\psi_{21}^{\left(2\right)}$			−0.066		−0.0058		−0.034
**Log likelihood**			−250.4		−180.3		−83.00

Notes: *** *p* < 0.01, ** 0.01 ≤ *p* ≤ 0.05.

For the multi-output technology function, previous table on the left, an increment of the factor unittype represents a 21% increment in the combined response, confirming the large impact that the type of service unit has. An increment in the factor donors represents 10% increment in the combined response. On the other hand increments in the number of donors above 70 years of age, represented by the factor donors 70–100, represent a 4% decrease in the combined response of kidney and liver transplants.

The random effect of level 3, representing variations at a hospital level follows a distribution of the form $\xi_{1j}^{\left(3\right)}∽N\left(0;0.17\right)$. The random effect of level 2, corresponding to the effect of the factor unittype in each hospital follows a distribution of the form $\xi_{2j}^{\left(2\right)}∽N\left(0;0.0026\right)$.

#### 4.1.2. Technical Efficiency Analysis

In determining the technical efficiency corresponding to the technology function (XZ) this paper assumes an output orientation as in the considered cased inputs such as the number of hospitals or population are not freely disposable.

For the technology function (4) the output-oriented technical efficiency is given by the power of the random effect ${\text{ζ}}_{1\text{k}}^{\left(3\right)}$ which represents the contribution to the output that is explained by neither the inputs nor external factors, refer to the following expression:

(5) $$\mu_{ij}=e^{{\text{ζ}}_{1\text{k}}^{\left(3\right)}}*e^{\left(\beta_{1}+\beta_{2}x_{2i}+\beta_{3}x_{3ik}+\cdots ++{\text{ζ}}_{2\text{k}}^{\left(2\right)}x_{2ik}\right)}$$

This random effect therefore represents the “efforts” conducted internally by the organization to maximize the outputs conditioned to given inputs and external factors. It is also possible to consider this random effect as a latent variable representing intrinsic characteristics of the hospital which are revealed via the observed outputs, *i.e.*, kidney and liver transplants performed.

The computation of the random effect, and subsequently the technical efficiency, is omitted for clarity purposes, interested readers may refer to [65]. The Table 3 presents the mean and the variance of the two random effects: (1) random-intercept, *i.e.*, technical efficiency and (2) random-coefficient.

**Table 3:** Technical efficiency corresponding to liver and kidney transplants.

Hospital	Random-Intercept (Technical Efficiency)		Random-Coefficient	
	Mean	Variance	Mean	Variance
**1**	−0.54019696	0.25949499	0.21117474	0.10144224
**2**	−0.06833701	0.33490417	0.02671442	0.13092133
**3**	0.2812933	0.34807859	−0.10996367	0.13607149
**4**	−0.31050514	0.25101169	0.12138321	0.09812593
**5**	0.2531918	0.21514489	−0.09897818	0.08410482
**6**	0.56289722	0.16580095	−0.22004877	0.0648152
**7**	0.45606749	0.18930796	−0.1782867	0.0740046
**8**	−0.2067746	0.24271143	0.08083269	0.09488118
**9**	−0.11436051	0.23453519	0.04470601	0.09168491
**10**	−0.13384933	0.21404345	0.05232462	0.08367424
**11**	−0.17941197	0.24079713	0.07013604	0.09413284

**Figure 4:**
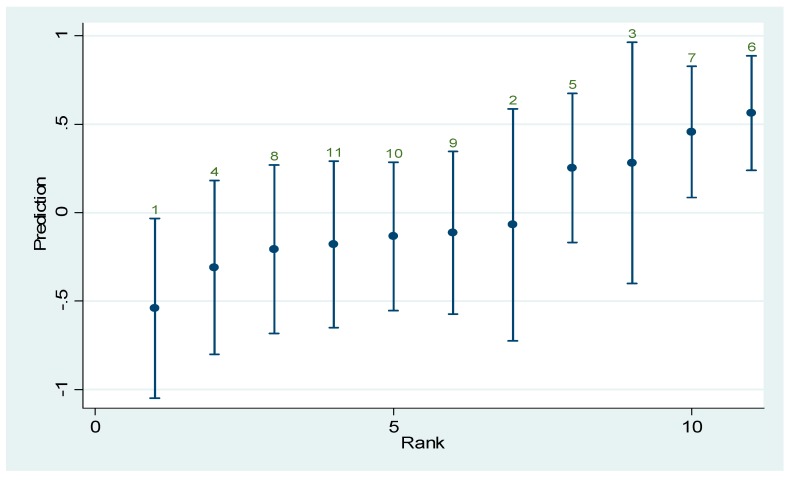
The technical efficiency for the considered hospitals.

Figure 4 provides a graphical representation of the technical efficiency achieved by hospitals considered, each dot representing the mean of the technical efficiency for the considered period (2008–2010) with its associated confidence interval.

According to Figure 4 we identify three different clusters of hospitals according to the technical efficiency achieved: (1) best performers: hospitals {#6,#7,#3,#5}, (2) average performers: hospitals {#2, #9, #10, #11, #8}, (3) low performer: region {#1,#4}.

For the multi-output technology function (4) the random coefficient in this case is associated with the factor *unittype*, therefore this random coefficient reflects economies of scale which may arise from an increase in the factor. Based on the mean of the random coefficient in Table 2 hospitals #1 and #4 would clearly benefit from an upgrade in their internal service units, e.g., deploying advanced neurological diagnosis.

### 4.2. Qualitative Analysis Based on Baldrige Constructs and Technical Efficiency

#### 4.2.1. Organizational Routines and Observed Performance

The previous section provided a ranking of the best hospitals according to their ability to maximize outputs (*i.e.*, kidney and liver transplants) subject to some levels of inputs (e.g., number of donors) and constrains (e.g., age of donors).

In order to investigate on the relationship between organizational routines within service units and the resulting performance this section models each service unit in terms of the Baldrige constructs (*i.e.*, leadership, strategic planning, customer focus, knowledge management, workforce focus and operation focus). In order to do so a thorough analysis based on the study of protocols implemented in each hospital, existing processes, quality certifications and external auditing has been conducted. Finally these data is triangulated with interviews with service managers in charge of the hospitals under study.

Based on the analysis of previous sources of information and following the Baldrige methodology levels for each construct are defined. Table 4 depicts the levels achieved by each hospital on each Baldrige construct along with the total Baldrige index (over a theoretical maximum of 550 points).

It is remarkable that all of the 11 service units considered manage to achieve excellent levels of quality as measured by the Baldrige index. Hospitals 2,5,6,7 and 9 are at the top, mostly due to their prominence in leadership and strategy.

**Table 4:** Levels of quality achieved in each hospital according to the Baldrige index.

Baldrige		Leader Ship			Strategic Planning		Focus Customer/Collaborators		Knowledge Management		Focus in Medical Staff		Focus in Operations	
ID			Leadership Unit Director	Model of Governance	Development of Strategy	Implementation on of the Strategy	Information on Customers & Collaborators	Customer and Collaborators Satisfaction	Performance Measurement	Management of the Information	Trust and Mutual Support	Motivation for the Reaching of Objectives	Design and Management of the Service Units	Operative Processes
1	394		50	80	65	65	80	80	90	70	65	80	75	70
2	478.75		90	95	90	90	90	90	95	95	80	80	75	70
3	476.5		90	95	90	90	90	90	95	90	80	80	75	70
4	407.5		60	80	65	65	80	80	90	80	70	80	75	70
5	478.75		90	95	90	90	90	90	95	95	80	80	75	70
6	478.75		90	95	90	90	90	90	95	95	80	80	75	70
7	478.75		90	95	90	90	90	90	95	95	80	80	75	70
8	448.5		80	95	80	80	85	80	90	90	70	80	75	70
9	478.75		90	95	90	90	90	90	95	95	80	80	75	70
10	446,25		80	95	80	80	85	80	90	85	70	80	75	70
11	403,25		60	80	70	65	80	75	85	80	65	80	75	70

As a preliminary analysis of the relationship between the technical efficiency achieved by each service unit, refer to section 2 and their organizational routines, the following Table 5 represents a conventional regression analysis in which technical efficiency is regressed over the Baldrige index.

**Table 5:** Technical efficiency corresponding to liver and kidney transplants.

	Input Variable	Coef.	Std. Err.	T	*p* > |t|	95% Conf. Int.
**Outcome: Technical efficiency**	Baldrige Index	0.006	0.001	5.72	0.000	(0.0036,0.0084)
	Constant Term	−2.733	0.4809	−5.68	0.000	(−3.821,−1.645)
**R-squared: 0.784**	**Prob** > **F: 0.0003**					

The model explains 78% of the variability of the problem (R-squared 0.78). We see that the variable Baldrige positively moderates the technical efficiency achieved and is statistically quite significant. (Coef: 0.006, *p*-value: 0.000). For illustration purposes a 10% increment in the level of leadership means a 0.072 increment in the technical efficiency achieved according to the model. The results show that high levels of organizational routines and processes positively moderate the resulting technical efficiency.

#### 4.2.2. Causal Relationships between Organizational Routines and Technical Efficiency

In order to further investigate the influence of each Baldrige indicator in the observed technical efficiency we conduct a multivariate factor analysis combined with a varimax rotation.

Based on the Table 6, we conclude that the dimensionality of the problem may be reduced down to two factors according to the proportion of variability explained (*i.e.*, 0.957 and 0.042 for factor 1 and factor 2, respectively).

**Table 6:** Multivariate factor analysis.

Factor	Eigenvalue	Difference	Proportion	Cumulative
Factor 1	5.356	5.116	0.957	0.9571
Factor 2	0.239	0.193	0.042	1.000
Factor 3	0.046	0.038	0.008	1.008
Factor 4	0.007	0.020	0.001	1.009
Factor 5	−0.012	0.029	−0.002	1.007
Factor 6	−0.041	0.0	−0.007	1.000
**LR test: independent *vs.* saturated**		**chis2(15) = 111.55**	**Prob > chi2 = 0.0000**	

**Figure 5:**
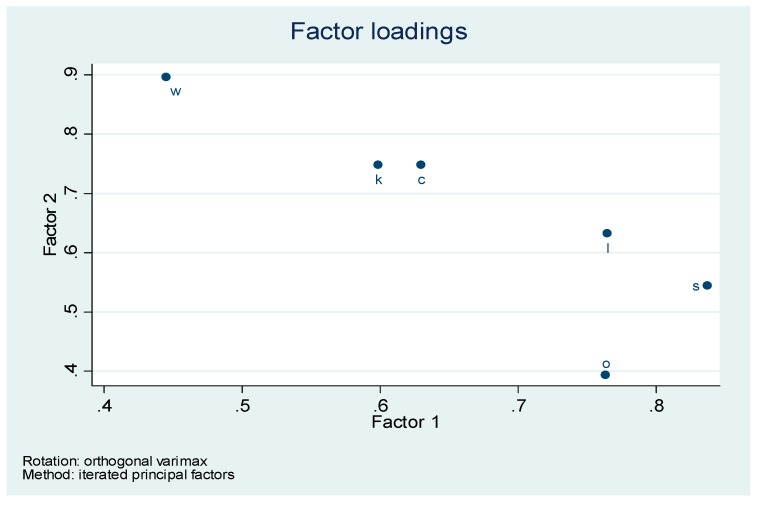
Factor loadings associated with factor 1 and factor 2.

Therefore retaining only two factors and performing a varimax rotation gives the factor loadings corresponding to the initial variables (*i.e.*, Baldrige constructs) as represented in Table 7 and Figure 5.

According to the Figure 5 we observe that factor 1 measures the level intensity of indicators: “leadership, strategy and operations focus”. Whereas factor 2 measures the level intensity of indicators: “customer focus, workers focus and knowledge management”. Combining the data (Table technical efficiency) with the data (varimax rotation) gives the following results (Table 8).

**Table 7:** Varimax rotation retaining two main factors.

Variable	Factor 1	Factor 2	Uniqueness
Leadership (l)	0.764	0.632	0.015
Strategic planning (s)	0.837	0.544	0.0028
Customer focus (c)	0.629	0.748	0.0436
Knowledge manag. (k)	0.599	0.748	0.081
Workforce focus (w)	0.445	0.896	−0.0014
Operations focus (o)	0.763	0.393	0.2621

**Table 8:** Technical efficiency (varimax rotation).

Technical Efficiency	Factor 1	Factor 2	
−0.540197	−1.365.368	−1.133.234	Level 1 low, level 2 low
−0.3105052	−2.428.829	0.368478	
−0.2067746	0.5289063	−0.5087735	Level 1 high, level 2 low
−0.179412	0.2368888	−2.163.429	
−0.1338493	0.5532684	−0.884609	
−01143605	0.0794354	0.8061788	Level 1 low, level 2 high
−0.068337	0.0794354	0.8061788	
0.2531918	0.3989328	0.583496	High levels in factors 1 and 2
0.2812933	0.239393	0.7124175	
0.4560675	0.55121	0.7432994	
0.5628972	1.126726	0.3016275	

Factor 1 measures leadership, strategy and operations.

Factor 2 measures the focus in the medical staff, the customer/collaborators and knowledge management.

The results show that:

1. To achieve excellence, the hospitals must show excellence in both dimensions.
2. The units showing high levels in the second dimension could easily improve by putting their efforts in dimension 1.
3. In a similar way, there are units showing high levels in the dimension 1 that are penalized by a low level in factor 2.
4. Showing low levels in both dimensions implies achieving very low levels in the technical efficiency domain.

## 5. Discussion, Conclusions and Future Areas of Research

Two have been the main purposes of this paper: (1) provide formal methods to conduct quantitative analysis of technical efficiency in organ transplant and (2) further investigate the impact of internal processes within organ transplant units in the observed performance.

Organ transplant systems present two important characteristics: interdependence and capacity of adaptation. Both characteristics make the management and evaluation of these systems complex. The present work has taken as an example of this complex healthcare system the donation and transplant system in a region of Spain.

This paper adopts a mixed method research since it combines in the research process elements of qualitative and quantitative research in order to achieve breadth and depth of understanding and validation. By considering the barriers observed in parametric models, this paper follows a parametric multilevel modeling approach to calculate the technical efficiency reached in donation and transplant healthcare service delivery system.

An initial quantitative exploration based in the technical efficiency concept is followed by several qualitative analyses aimed at explaining in more depth the mechanisms underlying the phenomena under observation. For this second part, concepts coming from the relational coordination framework and the Baldrige quality model have been applied. From the reviewed literature and the results obtained in the present work it is possible to argue that the excellence in the offering of services in donation and transplant requires: (1) excellence in the management of operational processes and (2) putting into action relational coordination mechanisms amongst the systems involved.

In relation to both components, the operational and relational one, to achieve excellence in healthcare delivery systems, it is possible to implement in practice mechanisms for the measurement of both components by making use of mixed methods, including both quantitative and qualitative tools. This way, the operational component is estimated by making use of the technical efficiency and the relational one by making use of the Baldrige indexes.

An important conclusion from this work is the existent relation prooved between the levels related to the Baldrige indexes and the technical efficiency observed in the donor and transplant units in the 11 analyzed hospitals in this study. This way it is possible to conclude how high levels in the Baldrige indicators become a necessary condition to get a high level in the service delivery.

As future areas of research we could consider to widen the simple to other health regions to analyze dependencies to a regional level and identify quantitative relationships amongst Baldrige indicators and the observed technical efficiency in other kind of transplants. Having more data could enable the development of detailed analysis about the importance of each Baldrige construct.

## Appendix

**Table A1:** Panel data corresponding to 11 hospitals involved in the Spanish system for organ donation and transplant for the period (2008–2010) [62].

Hospital ID	Type of Unit	Donors	Donors above 70	Year	Kidney Transplants	Liver Transplants
1	0	6	2	2008	10	1
		0	0	2009	0	0
		3	1	2010	6	2
		0	0	2011	0	0
2	1	17	3	2008	32	16
		21	8	2009	32	20
		22	7	2010	30	19
		21	11	2011	26	20
3	1	11	1	2008	20	9
		13	3	2009	20	12
		18	7	2010	30	13
		24	14	2011	42	22
4	0	3	2	2008	6	3
		3	1	2009	6	2
		2	0	2010	4	0
		1	1	2011	2	1
5	0	2	2	2008	2	4
		3	2	2009	4	2
		4	3	2010	8	4
		7	6	2011	14	7
6	2	16	5	2008	26	15
		19	9	2009	26	17
		26	11	2010	46	26
		26	11	2011	41	25
7	0	10	5	2008	16	10
		5	2	2009	8	5
		3	0	2010	6	3
		6	6	2011	10	6
8	0	3	1	2008	4	3
		3	2	2009	4	3
		2	1	2010	4	2
		4	4	2011	4	4
9	2	13	4	2008	24	13
		9	4	2009	14	9
		5	2	2010	10	4
		8	2	2011	14	7
10	2	13	3	2008	24	13
		11	4	2009	18	11
		14	3	2010	26	12
		7	1	2011	12	7
11	0	6	4	2008	7	6
		0	0	2009	0	0
		4	3	2010	8	4
		2	2	2011	2	2
